# Isothiocyanatostilbenes as novel c-Met inhibitors

**DOI:** 10.18632/oncotarget.5748

**Published:** 2015-10-31

**Authors:** Alana L. Gray, David T. Coleman, Reneau F. Castore, Mohamed M. Mohyeldin, Khalid A. El Sayed, James A. Cardelli

**Affiliations:** ^1^ Louisiana State University Health Sciences Center - Shreveport, Shreveport, LA, USA; ^2^ Feist-Weiller Cancer Center, Shreveport, LA, USA; ^3^ University of Louisiana - Monroe, Monroe, LA, USA

**Keywords:** c-Met, isothiocyanatostilbenes, DIDS, H2DIDS

## Abstract

The hepatocyte growth factor receptor (HGFR or c-Met) is a driver of multiple cancer subtypes. While there are several c-Met inhibitors in development, few have been approved for clinical use, warranting the need for continued research and development of c-Met targeting therapeutic modalities. The research presented here demonstrates a particular class of compounds known as isothiocyanatostilbenes can act as c-Met inhibitors in multiple cancer cell lines. Specifically, we found that 4,4′-Diisothiocyanatostilbene-2,2′-disulfonic acid (DIDS) and 4,4′-Diisothiocyanatodihydrostilbene-2,2′-disulfonic acid (H2DIDS) had c-Met inhibitory effective doses in the low micromolar range while 4-acetamido-4′-isothiocyanatostilbene-2,2′-disulfonic acid (SITS) and 4,4′-dinitrostilbene-2, 2′-disulfonic acid (DNDS) exhibited IC_50_s 100 to 1000 fold higher. These compounds displayed much greater selectivity for inhibiting c-Met activation compared to similar receptor tyrosine kinases. In addition, DIDS and H2DIDS reduced hepatocyte growth factor (HGF)-induced, but not epidermal growth factor (EGF)-induced, cell scattering, wound healing, and 3-dimensional (3D) proliferation of tumor cell spheroids. In-cell and cell-free assays suggested that DIDS and H2DIDS can inhibit and reverse c-Met phosphorylation, similar to SU11274. Additional data demonstrated that DIDS is tolerable *in vivo*. These data provide preliminary support for future studies examining DIDS, H2DIDS, and derivatives as potential c-Met therapeutics.

## INTRODUCTION

The c-Met receptor tyrosine kinase has been demonstrated to be sufficient for oncogenic transformation [1], is a major regulator of invasive growth [2], and is a key contributor to tumor progression [3–5]. Therefore, it is not surprising that c-Met is an established therapeutic target for multiple types of cancers [reviewed in [6]]. Studies have shown that targeting the c-Met pathway can prevent and, in some cases, even reverse advanced stages of tumor progression as evidenced by a reduction in the number and size of metastatic lesions [7]. While several tyrosine kinase inhibitors (TKIs) have been identified as highly effective anti-cancer therapies, they are often prohibitively toxic and it is also common for patients to develop an acquired resistance to these drugs [8, 9]. Because of this, there is a continued need for the development of an expanded repertoire of TKI inhibitors.

A recent search of http://ClinicalTrials.gov revealed over 50 ongoing studies examining the role of c-Met in cancer. However, current FDA-approved c-Met inhibitors include only crizotinib (Xalkori; Pfizer) and cabozantinib (Cometriq; Exelixis). Both of these compounds are non-specific c-Met inhibitors, such that crizotinib also targets ROS proto-oncogene 1, receptor tyrosine kinase (ROS1) and anaplastic lymphoma kinase (ALK) while cabozantinib inhibits vascular endothelial growth factor receptor 2 (VEGFR2), in addition to c-Met.

The c-Met pathway has been shown to be involved in normal homeostatic functions. However, dysregulation of c-Met signaling caused by receptor mutation, c-Met or HGF overexpression, gene amplification, or transcriptional upregulation can result in various pathological outcomes, including cancer [10–13]. Activation of the c-Met receptor classically occurs following binding of its only known ligand, hepatocyte growth factor (HGF) prior to c-Met dimerization. This dimerization triggers multiple autophosphorylation events of the cytoplasmic tail of c-Met that are required for propagation of downstream signaling leading to increased cell proliferation, motility, and tumor cell invasion and metastasis. Multiple approaches targeting aberrant signaling through the c-Met pathway have been employed including anti-HGF and anti-c-Met antibodies, HGF and c-Met competitive antagonists, and inhibitors of signaling molecules downstream of c-Met activation [reviewed in [12]]. Our research has uncovered a class of compounds known as isothiocyanatostilbenes that act as c-Met inhibitors. We believe these compounds could serve as templates for the development of structurally similar and more efficacious anti-c-Met therapeutic agents.

## RESULTS

### Isothiocyanatostilbenes reduce c-Met phosphorylation

**Figure 1:**
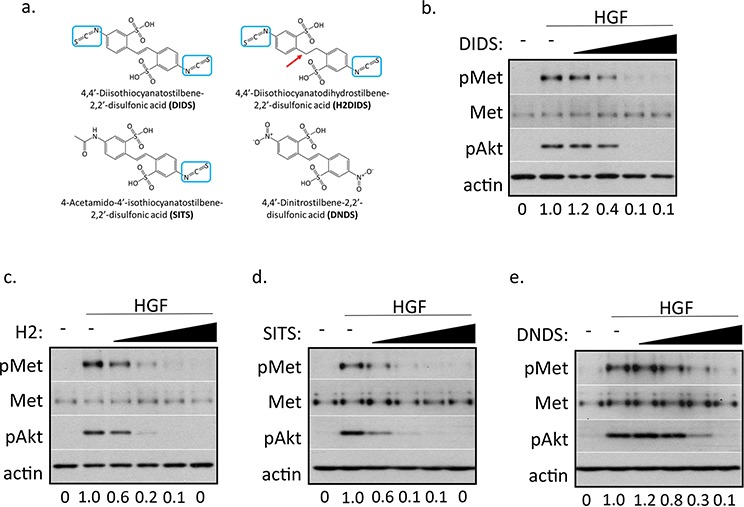
Stilbene analogs reduce c-Met activation in a dose-dependent manner **a.** Chemical structures of the investigated stilbene analogs. All compounds share the same stilbene core, except H2DIDS which is lacking a double bond linker of both aryl groups, indicated by a red arrow. Three of the four compounds contain at least one *p*-isothiocyanate group, indicated by blue boxes. **b–e.** DU145 cells were treated with 33 ng/ml HGF for 20 minutes in the presence of 500 nM, 2 μM, 8 μM, 32 μM DIDS (b) or H2DIDS (c), 125 μM, 250 μM, 500 μM, 1 mM SITS (d), or 250 μM, 500 μM, 1 mM, 2 mM DNDS (e). Western blot was used to analyze the indicated proteins. Densitometry shows changes in pMet compared to HGF control normalized to 1.

While using DIDS as an inhibitor of anion exchangers [14, 15], by happenstance, we found it potently inhibited c-Met phosphorylation. Importantly, DIDS reduced activation of c-Met at concentrations that were too low to inhibit anion exchangers. This indicated that DIDS may act as a c-Met inhibitor independent of its ability to target anion transport. The ability of DIDS to inhibit c-Met activation at a reasonably low concentration suggested DIDS and other stilbene analogs may be potential clinical therapeutics. We subsequently obtained additional stilbene compounds, H2DIDS, SITS, and DNDS, to assess their structure activity relationship. These compounds have similar structural scaffolds and differ in the number of isothiocyanate substituent groups (Figure 1a). DNDS is structurally similar, but the two isothiocyanate substituents were replaced by nitro groups. Notably, H2DIDS only differs from DIDS by its reduced two-carbon linker chain that connects both aromatic rings; therefore, H2DIDS is the dihydro analog of DIDS. Western blot analysis of a serum-free dose response curve in DU145 prostate cancer epithelial cells showed that ~8 μM DIDS reduced c-Met activation and downstream Akt by ~90% (Figure 1b). Figures 1c–1e show that H2DIDS, SITS and DNDS inhibited c-Met by ~90% at 8 μM, 250 μM and 2 mM, respectively. These compounds also exhibited similar, though not identical, effective doses in PC3 prostate cancer (Figure S1a), MDA-MB-231 breast cancer (Figure S1b), and HCC1806 breast cancer (Figure S1c) cells. In all tested cell lines, DIDS and H2DIDS consistently demonstrated higher potencies for inhibiting c-Met activation by HGF; therefore, we focused on these two compounds for the remaining duration of the study. We found that DIDS and H2DIDS had slightly higher effective concentrations in dose response assays containing 10% FBS (Figure S2a and S2b).

**Figure 2:**
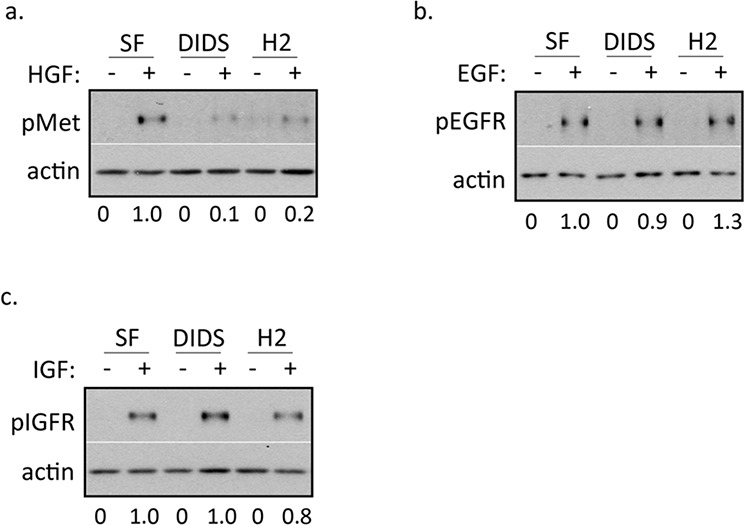
DIDS and H2DIDS reduce activation of c-Met, but not EGFR or IGFR DU145 cells were treated with 33 ng/ml HGF **a.** 100 ng/ml EGF **b.** or 100 ng/ml IGF **c.** for 20 minutes in the presence of 4 μM DIDS or 25 μM H2DIDS. Western blot was used to analyze the indicated proteins. Densitometry shows changes in pMet compared to HGF control normalized to 1.

To begin to assess the specificity of DIDS and H2DIDS, we treated DU145 cells with concentrations of DIDS and H2DIDS known to inhibit c-Met activation in combination with HGF, epidermal growth factor (EGF), or insulin-like growth factor (IGF). Western blot analysis revealed that concentrations of DIDS and H2DIDS that reduce c-Met activation do not significantly reduce EGFR or IGFR activation (Figure 2a–2c), suggesting some degree of selectivity for c-Met.

### DIDS and H2DIDS reduce HGF-induced cell motility and invasion

**Figure 3:**
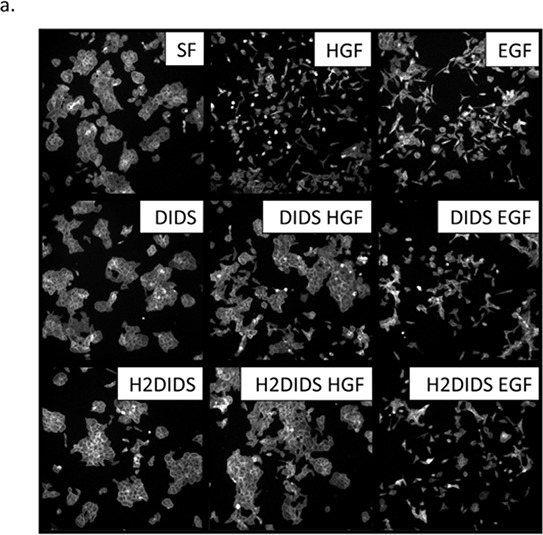

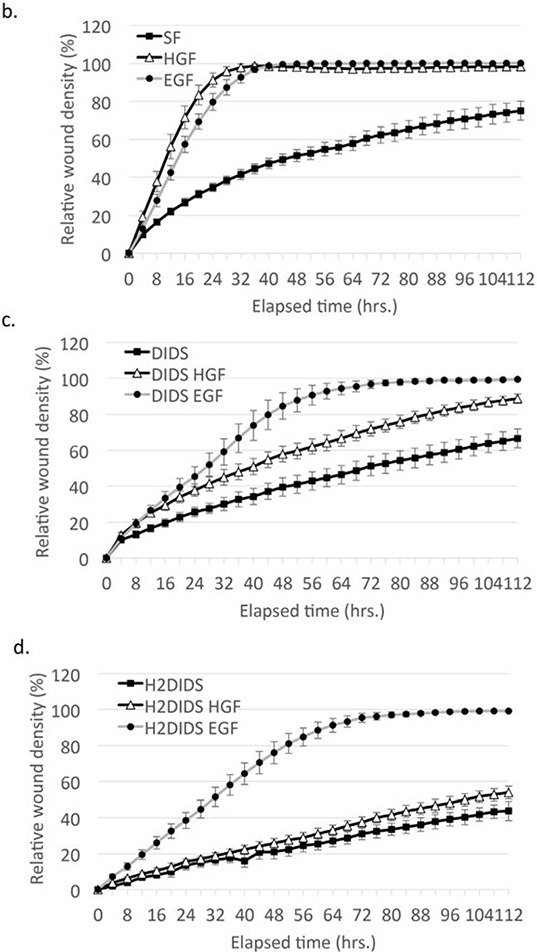
DIDS and H2DIDS reduce HGF-induced but not EGF-induced cell scattering and wound healing **a.** DU145 cells were treated with 33 ng/ml HGF or 100 ng/ml EGF overnight in the presence of 4 μM DIDS or 25 μM H2DIDS. Cells were fixed and stained for actin. Representative images are shown. **b–d.** Confluent monolayers of DU145 cells were wounded prior to treatment with 33 ng/ml HGF or 100 ng/ml EGF for the indicated times in the presence of 4 μM DIDS or 25 μM H2DIDS. Data are shown as mean ± S.E.M; n = 3.

**Figure 4:**
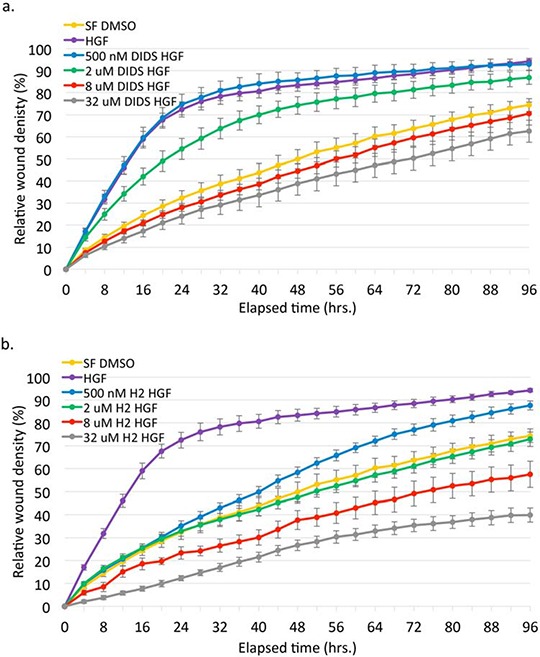

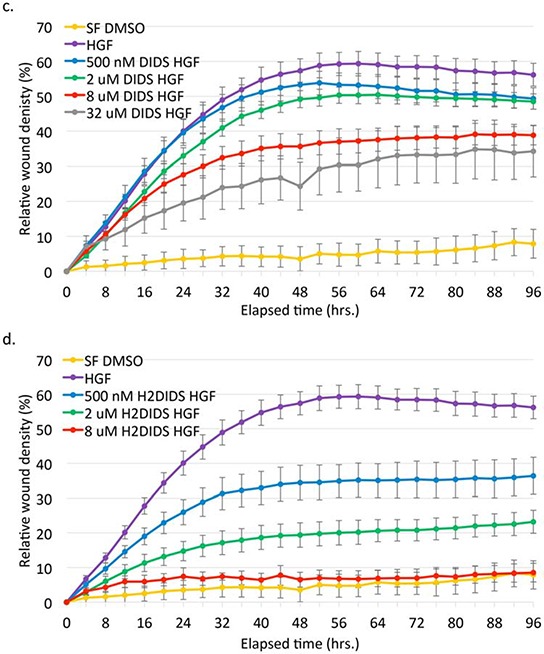
DIDS and H2DIDS reduce HGF-induced wound healing and invasion in a dose-dependent manner DU145 cells were treated with the indicated concentrations of DIDS **a, c.** or H2DIDS **b, d.** in the presence of 33 ng/ml HGF. For invasion (c, d) wounded cells were overlaid with Matrigel diluted 1:5 in serum-free media. Data are shown as mean ± S.E.M.; n = 3.

We next sought to determine the effect of these compounds on HGF-mediated phenotypes. DU145 cells exhibit a striking scattered and motile phenotype in response to EGF and HGF stimulation indicative of an epithelial-mesenchymal transition. An initial dose response scattering assay revealed that DIDS inhibited HGF-induced scattering between 1.8 μM and 8 μM and H2DIDS was effective between 8 μM and 40 μM (Figure S3). Using concentrations of DIDS and H2DIDS within this range, we discovered that these same concentrations were able to reduce HGF-induced, but not EGF-induced cell scattering (Figure 3a). This was consistent with our observations that DIDS and H2DIDS do not inhibit EGFR signaling (Figure 2b). Similarly, HGF-induced wound healing, a unique collective form of cell motility, was also significantly reduced while the same concentrations had minimal effects on EGF-mediated wound healing (Figure 3b–3d). We further examined dose response effects of DIDS and H2DIDS on c-Met phenotypes. Figure 4a and 4b show that DIDS and H2DIDS reduce HGF-induced wound healing in a dose response manner.

Similarly, these compounds inhibited HGF-induced invasion through Matrigel in a dose-dependent manner (Figure 4c and 4d). Although seemingly effective, the highest concentration of H2DIDS is not shown in Figure 4d due to a consistent but anomalous precipitant or interaction of H2DIDS with the Matrigel that resulted in grainy images precluding accurate quantitative analysis. DIDS was able to inhibit wound healing more effectively than similar concentrations used to inhibit invasion. H2DIDS appeared to be slightly more effective than DIDS in wound healing and much more effective in invasion assays. Notably, these concentrations of DIDS and H2DIDS had minimal effects on serum-free proliferation (Figure S4a and S4b) and proliferation in complete media (Figure S4c and S4d) in a 2D assay. Collectively, these data suggest that DIDS and H2DIDS can negatively affect c-Met/HGF-induced cell motility and invasion, but not by affecting proliferation in 2D.

### DIDS and H2DIDS decrease HGF-induced 3D spheroid proliferation

**Figure 5:**
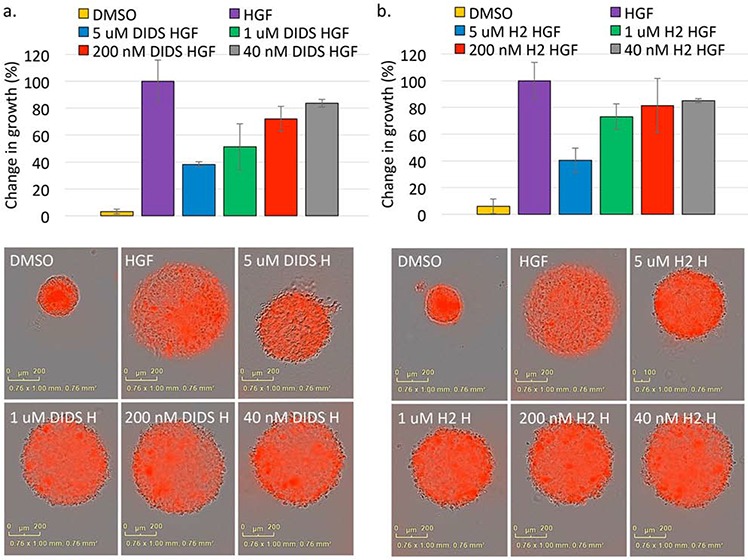
Stilbene compounds reduce 3D spheroid growth DU145 spheroids suspended in Matrigel were treated with DIDS **a.** or H2DIDS **b.** in the presence of 33 ng/ml HGF for 72 hours. Data are shown as the mean percent change in growth from T = 0 to T = 72 hours ± S.E.M.; *n* = 3. Representative images at the 72 hour timepoint are shown.

In an attempt to better determine effective concentrations that may be useful for *in vivo* applications, we analyzed the effects of these stilbenes on cells grown in a 3D environment. For this assay, cells were embedded in Matrigel and treated concomitantly with DIDS or H2DIDS. Dose response curves showed that 5 μM DIDS and H2DIDS decreased HGF-induced DU145 spheroid growth by ~60% and still had some inhibitory effects as low as 40 nM (Figure 5a and 5b). These data suggest that DIDS and H2DIDS are effective in environments that more closely mimic *in vivo* conditions.

### DIDS and H2DIDS inhibit and reverse c-Met phosphorylation

**Figure 6:**
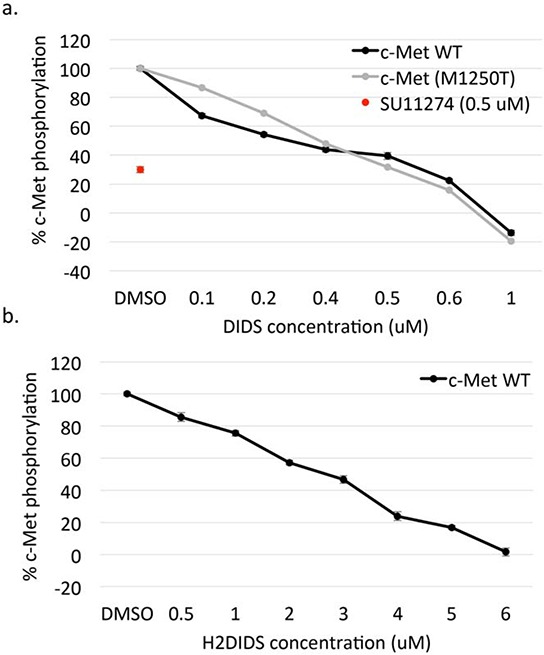

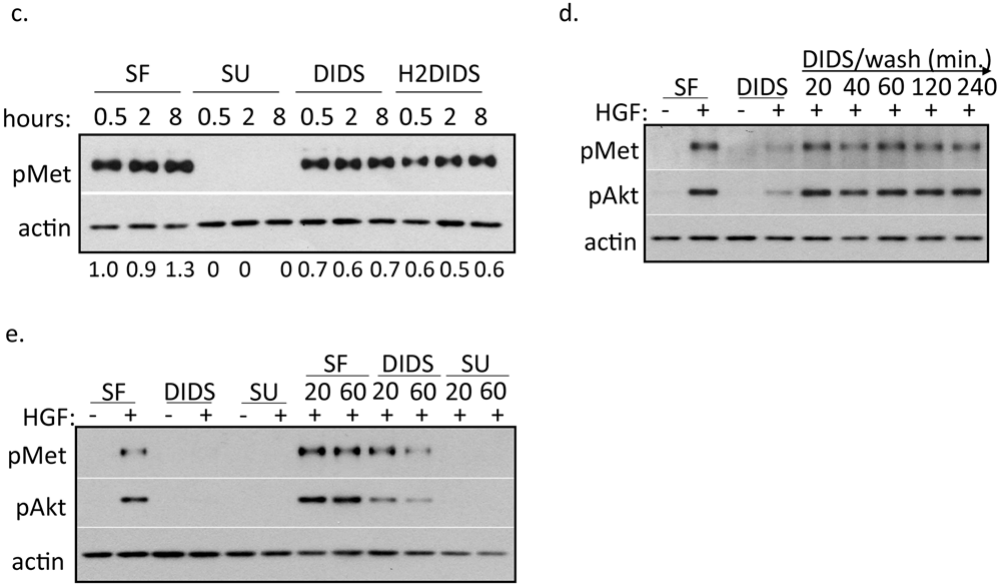
DIDS and H2DIDS inhibit and reverse c-Met phosphorylation Inhibition of wild-type (WT) and mutant (M1250T) c-Met phosphorylation was examined using various concentrations of DIDS **a.** or H2DIDS **b.** Data are shown as mean ± S.E.M.; *n* = 3. **c.** H1993 cells were treated with 5 μM SU11274, 5 μM DIDS, 5 μM H2DIDS, or serum-free media for the indicated times. Western blot was used to analyze pMet expression. **d.** DU145 cells were treated with 4 μM DIDS for 20 minutes prior to washing for the indicated times followed by treatment with 33 ng/ml HGF for 20 minutes. **e.** DU145 cells were treated with 33 ng/ml HGF for 20 minutes prior to washing followed by 4 μM DIDS or 10 μM SU11274 for 20 or 60 minutes. DIDS and SU11274 were treated with HGF for 20 minutes as a control. Western blot was used to analyze the indicated proteins. Densitometry shows changes in pMet compared to HGF control normalized to 1.

We next sought to better determine the mechanism by which isothiocyanatostilbenes inhibit c-Met phosphorylation. SU11274 is a well-established class I c-Met inhibitor that competitively binds the ATP-binding site of c-Met [16, 17]. In order to determine if DIDS acts in a manner similar to a class I c-Met inhibitor, we compared DIDS and H2DIDS to SU11274 in the following assays. First, *ex vivo* kinase assays were performed. At 500 nM, SU11274 reduced c-Met phosphorylation by ~70% and DIDS was found to reduce activation of wild-type c-Met with an IC_50_ of 300 nM (Figure 6a). H2DIDS was not as effective as DIDS as the IC_50_ for H2DIDS was 3.6 μM (Figure 6b). We also tested the ability of DIDS to inhibit c-Met M1250T (M1268T), a known mutant form of the receptor found in several types of cancers that can increase kinase activity and alter substrate specificity [18]. Similar to SU11274 [19], DIDS inhibited this form of the receptor with an IC_50_ of 370 nM (Figure 6a).

Although it appeared DIDS can act as an ATP-binding pocking inhibitor, we further examined other possible mechanistic avenues. H1993 lung cancer cells were treated with DIDS, H2DIDS, and SU11274 prior to western blot analysis. H1993 cells have c-Met amplification such that they have high levels of pMet, even in the absence of HGF, due to constitutive c-Met dimerization and autophosphorylation [20, 21]. Interestingly, SU11274 ameliorated pMet levels in H1993 cells while DIDS and H2DIDS had much less significant effects (Figure 6c). These data suggested that DIDS and H2DIDS act in a similar manner to SU11274 in their ability to inhibit c-Met kinase activity; however, SU11274 is able to ameliorate c-Met activation occurring independent of ligand-receptor interaction while DIDS and H2DIDS are much less effective.

It has been reported in the literature that DIDS can exhibit covalent crosslinking properties [22, 23]. To determine if this could account for c-Met inhibition, we treated DU145 cells with DIDS followed by one wash step and up to 1 hour recovery time prior to activation with HGF. Even as early as 20 minutes post-wash, HGF was able to activate c-Met to non-DIDS treated levels (Figure 6d) suggesting DIDS does not irreversibly bind c-Met. Surprisingly, we found that DIDS treatment post HGF treatment reduced pMet levels suggesting that DIDS is able to reverse c-Met activation under certain conditions (Figure 6e). This has also been observed with SU11274 previously [24] and is further supported by our data (Figure 6d).

### DIDS is well-tolerated in mice

**Figure 7:**
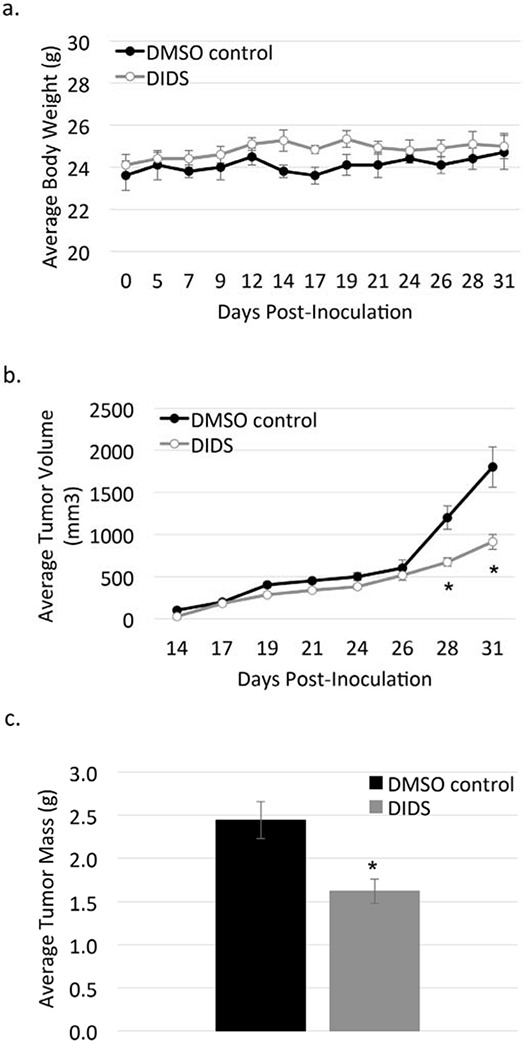
DIDS is not toxic and may reduce tumor progression *in vivo* Mice were treated with 5 mg/kg DIDS or equivalent volume DMSO for 14 days, followed by 10 mg/kg DIDS for an additional 16 days. **a.** Body weight of treated mice was measured throughout the duration of the experiment. **b.** Caliper measurements were taken on the indicated days and average tumor volume was calculated. **c.** Tumors were harvested at the end of the experiment and weighed. Data are shown as mean ± S.E.M.; *n* = 1; *p < 0.05.

Since DIDS and H2DIDS were capable of inhibiting c-Met activation at relatively low concentrations and no previous *in vivo* data on these compounds has been reported to our knowledge, we performed a preliminary experiment in a mouse model of breast cancer to determine if DIDS could be tolerated *in vivo*. For ~2 weeks, mice were treated with 5 mg/kg DIDS delivered intraperitoneally. No obvious signs of toxicity were observed, so DIDS treatments were increased to 10 mg/kg for ~2 additional weeks. Figure 7a shows that no change in body weight was observed in DIDS-treated mice compared to mice treated with DMSO control. Tumors became palpable on day 14 and measurements taken via caliper showed DIDS began to reduce tumor size towards the end of the experiment (Figure 7b). Differences in overall tumor weight were evident following necropsy (Figure 7c).

## DISCUSSION

The accumulation of information implicating c-Met as a major regulator of tumor progression has and continues to drive the search for effective c-Met inhibitors for over 30 years [25]. Here, we report the finding that isothiocyanatostilbenes are c-Met inhibitors. Notably, DIDS and H2DIDS have two isothiocyanate groups and were consistently more effective at c-Met inhibition than SITS, which has one isothiocyanate group while the other was replaced by an acetamido group, and DNDS, which has no isothiocyanate groups. Additionally, SITS was more effective at reducing c-Met phosphorylation than DNDS based on western blot analysis of multiple cancer cell lines (Figures 1 and S1). These data suggest that the isothiocyanate group is the principal bioactive moiety for c-Met inhibition.

Cell-free kinase assays suggested DIDS has a 300 nM IC_50_ for c-Met kinase activity while H2DIDS has an IC_50_ in the low micromolar range (Figure 3a and 3b). These data are supported by the findings of Bültmann and Starke initially implicating DIDS as an ATP-binding site inhibitor of the P_2X_ purinoceptor [26]. The reported IC_50_ of DIDS for the P_2X_ purinoceptor is similar to the IC_50_ for c-Met (Figure 3a). Additionally, DIDS and H2DIDS inhibited HGF-mediated phenotypes in various cell assays, but had minimal effects on EGF-mediated phenotypes (Figures 3, 4, 5). Finally, preliminary studies examining toxicity of DIDS in an animal model suggest DIDS is well-tolerated *in vivo* and may be anti-tumorigenic, even in a tumor model not primarily driven by c-Met (Figure 7). We predict that future studies examining DIDS efficacy in a c-Met driven tumor model may show greater anti-tumorigenic efficacy. Thus, the data presented herein identify isothiocyanatostilbene analogs as novel c-Met inhibitory leads in multiple model systems.

Isothiocyanatostilbenes are classically used as anion transport inhibitors [27–29], although other roles for these compounds have been reported. Specifically, DIDS has been demonstrated to inhibit protein translocation across the ER membrane [30] and inhibit matrix metalloproteinase release [31], both at concentrations of 400 μM and greater. At low micromolar concentrations, DIDS was found to prevent the interaction of human immunodeficiency virus type-1 (HIV-1) with CD4 T-cell receptors [32] and reduce activity of succinic dehydrogenase and F_0_F_1_-ATP synthase [33].

Specificity is desirable in the clinic to ensure predictability, and although others have shown DIDS can affect other targets, our results found DIDS to be tolerable in mice and selective for c-Met over other tested receptor tyrosine kinases. Furthermore, of the two FDA-approved c-Met inhibitors, cabozantinib is prescribed with c-Met as the intended primary target; whereas crizotinib is prescribed based on ALK expression status. This highlights the lack of specific c-Met inhibitors approved for use, and that it is likely c-Met inhibitors with additional targets will prove more successful than specific inhibitors. Multi-kinase inhibitors can be effective at preventing the acquisition of resistance pathways, and c-Met signaling as a resistance pathway is amply reported throughout the literature [34–38], making it an important secondary target for therapy as well.

Interestingly, DIDS is reported to target P_2X_, P_2Y_, and P_2Z_ receptors [26, 39–41] and recent large scale genetic screening data from Wilson *et al*. suggest that P_2Y_ receptor signaling is one mechanism that contributes to resistance to ALK inhibitors, such as crizotinib [42]. Based on this information, it's conceivable that combination therapy of crizotinib and DIDS may prevent P_2Y_ ALK inhibitor resistance while enhancing c-Met inhibition [43, 44], thus targeting two potential resistance pathways following crizotinib treatment.

We acknowledge the limitations of these compounds having possible promiscuous reactivity under particular conditions and recognize that the compounds tested within this study are likely not ideal candidates for therapeutic use; however, they can serve as scaffold templates to achieve improved specificity and pharmacologic profiles. Overall, these data identify isothiocyanatostilbenes as effective inhibitors of c-Met phosphorylation that are tolerable *in vivo*, based on studies using DIDS. Future studies will test the efficacy of these compounds and structurally similar derivatives toward c-Met-driven animal tumor models.

## MATERIALS AND METHODS

### Ethics statement

Investigation has been conducted in accordance with the ethical standards and according to the Declaration of Helsinki and according to national and international guidelines and has been approved by the authors' institutional review board.

### Cell culture

DU145, PC3, H1993 and HCC1806 cells were maintained in RPMI 1640 (Cellgro; Manassas, VA) supplemented with 10% fetal bovine serum (FBS) (Gemini; West Sacramento, CA). MDA-MB-231 cells were maintained in DMEM (Cellgro) supplemented with 10% FBS. All cells were obtained from American Type Culture Collection and grown at 37°C in 5% CO_2_.

### Materials

The stilbene compounds 4,4′-Diisothiocyanatostilbene- 2,2′-disulfonic acid (DIDS), 4-acetamido-4′-isothiocyanatostilbene-2,2′-disulfonic acid (SITS), 4,4′- dinitrostilbene-2,2′-disulfonic acid (DNDS) were purchased from Sigma-Aldrich (St. Louis, MO) and 4,4′-Diisothiocyanatodihydrostilbene-2,2′-disulfonic acid (H2DIDS) was purchased from Life Technologies (Grand Island, NY). SU11274 was purchased from EMD Millipore (Billerica, MA). Recombinant growth factors include: HGF (EMD Millipore), EGF (Sigma-Aldrich), and IGF (PeproTech; Rocky Hill, NJ).

### Western blot analysis

Cells were grown to ~70% confluency prior to treatment. Cells were serum-starved in serum-free media for 30 minutes prior to treatment in complete media or serum-free media for the indicated times. Cell lysates were collected in Laemmli (125 mM Tris, 4% SDS, 0.01% bromophenol blue, 30% sucrose) containing 0.5% β-mercaptoethanol and boiled ~8 minutes. Primary antibodies include: phospho-c-Met (Y1234/1235), phospho-Akt (S473), pEGFR (S845), pIGF-IRβ (Y1135/1136) (Cell Signaling Technology; Beverly, MA); α-actin (Sigma-Aldrich), c-Met (C-28) (Life Technologies). Secondary antibodies include: horseradish peroxidase-conjugated anti-rabbit and anti-mouse (GE Healthcare; Pittsburgh, PA). ECL 2 was used for chemiluminescent detection (Thermo Scientific; Rockford, IL). Densitometry was calculated using ImageJ (NIH).

### Wound healing and invasion

DU145 cells were grown to ~100% confluency prior to wounding with the IncuCyte™ WoundMaker™ (Essen Bioscience; Ann Arbor, MI) and washed once with complete media. For invasion, cells were covered with Matrigel diluted 1:5 in serum-free RPMI following wounding. Cells were treated with 4 μM DIDS or 25 μM H2DIDS with or without 33 ng/ml HGF or 100 ng/ml EGF for up to 4 days. Images were taken every 4 hours with the IncuCyte™ ZOOM imaging system (Essen Bioscience). Quadruplicate replicates were used in each experiment. Data are presented as percent wound density ± standard error of the mean (S.E.M.).

### Scattering assay

DU145 cells were grown to ~50% confluency. Cells were treated overnight with 4 μM DIDS or 25 μM H2DIDS in the presence or absence of 33 ng/ml HGF or 100 ng/ml EGF. Cells were fixed with 4% paraformaldehyde and stained with Oregon Green 488 phalloidin (Invitrogen; Carlsbad, CA). Images were acquired on an Eclipse TE300 inverted microscope (Nikon; Tokyo, Japan) with NIS Elements version 4.13.04 software. Presented images were taken using a 10X objective.

### Phosphorylation inhibition assay

Z'-LYTE™ Kinase Assay-Tyr6 Peptide kit (Invitrogen) was used to assess the ability of tested compounds to inhibit c-Met phosphorylation. Briefly, 20 μl/well reactions were set up in 96-well plates containing kinase buffer, 200 μM ATP, 4 μM Z-LYTE™ Tyr6 Peptide substrate, 2500 ng/ml c-Met kinase and DIDS or H2DIDS at various concentrations. After 1 hour incubation at room temperature, 10 μl development solution containing site-specific protease was added to each well. Incubation was continued for 1 hour. The reaction was then stopped, and the fluorescent signal ratio of 445 nm (coumarin)/520 nm (fluorescein) was determined on a plate reader (BioTek FLx800™), which reflects the peptide substrate cleavage status and/or the kinase inhibitory activity in the reaction. Appropriate controls were conducted to ensure that DIDS and H2DIDS did not interfere with the reaction or emit detectable fluorescence. The IC_50_ value for each compound was calculated by nonlinear regression of log concentration versus % c-Met phosphorylation ± S.E.M., implemented in GraphPad Prism version 5.0 (GraphPad Software, CA, USA).

### 2D proliferation

Cells were seeded to ~30% confluency prior to treatment with DIDS or H2DIDS at 0.5 μM, 2 μM, 8 μM, 32 μM, or 64 μM for 24 hours, 48 hours, or 72 hours in complete or serum-free RPMI. A T0 timepoint was also measured. For each timepoint, cells were exposed to Cell Titer Blue reagent (Promega) for 1 hour at 37°C 5% CO_2_. Experiments were performed with quadruplicate replicates. Fluorescence was measured using a BioTek Synergy 4 plate reader with Gen5 software.

### 3D spheroid proliferation

Prior to seeding, cells were incubated with 2.5 ng/μl CellTracker Red (Life Technologies) in complete DMEM lacking Phenol Red for 5 minutes at room temperature then centrifuged at 1000 RPM for 5 minutes. Dyed cells were resuspended in complete DMEM lacking Phenol Red and containing 5% Matrigel before adding 5 μM, 10 μM, 20 μM, or 40 μM DIDS or H2DIDS +/− HGF. The mixture of cells, treatments, and Matrigel in complete media was seeded at 2000 cells/well in a 96 well plate. The plate was centrifuged at 1000 RPM for 3 minutes to collect the cells into the bottom of the plate. Spheroid growth was imaged for up to 80 hours and analyzed for average red object area using the IncuCyte™ ZOOM software. Data are shown as percent change in spheroid growth between T0 and 80 hours with HGF normalized to 100% change in growth. Images were captured by the IncuCyte™ ZOOM imaging system and representative 10X images are shown.

### Xenograft studies

All animal experiments were approved by the Institutional Animal Care and Use Committee, University of Louisiana at Monroe, and were handled in strict accordance with good animal practice as defined by the NIH guidelines. Athymic nude mice (Foxn1^nu^/Foxn1^+^, 4–5 weeks, female) were purchased from Harlan (Indianapolis, IN). The mice had free access to standard pellet food and water. The animals were acclimated to animal house facility conditions at a temperature of 18–25°C, with a relative humidity of 55 to 65% and a 12 h light/dark cycle, for one week prior to the experiments. MDA-MB-231/GFP human breast cancer cells were cultured and resuspended in serum-free DMEM medium (20 μl). After anesthesia, cell suspensions (1 × 10^6^ cells/20 μl) were inoculated subcutaneously into the second mammary gland fat pad just beneath the nipple of each animal to generate orthotopic breast tumors. At 48 h post-inoculation, the mice were randomly divided into two groups: i) the vehicle-treated control group (*n* = 5), ii) the DIDS-treated group (*n* = 5). Treatment (3X/week) started 5 days post-inoculation with intraperitoneal (i.p.) administered vehicle control (DMSO/saline) or 5 mg/kg DIDS. The dose of DIDS was increased to 10 mg/kg on day 19 post-inoculation. The mice were monitored by measuring tumor volume, body weight, and clinical observation. Tumor volume (V) was calculated by V = L/2 × W^2^, where L was the length and W was the width of tumors. The results are presented as average ± S.E.M. Differences among various treatment groups were determined by the analysis of variance (ANOVA) followed by Dunnett's test using PASW statistics version 18. A difference of *P* < 0.05 was considered statistically significant as compared to the vehicle-treated control group.

## SUPPLEMENTARY FIGURES


