# Supplementary material for: Isothiocyanatostilbenes as novel c-Met inhibitors
Source: Oncotarget. 2015 Oct 31;6(38):41180–93. doi: 10.18632/oncotarget.5748 (PMC4747398; doi:10.18632/oncotarget.5748)
Supplement: Supplementary file 1 [file oncotarget-06-41180-s001.pdf]

SUPPLEMENTARY FIGURES

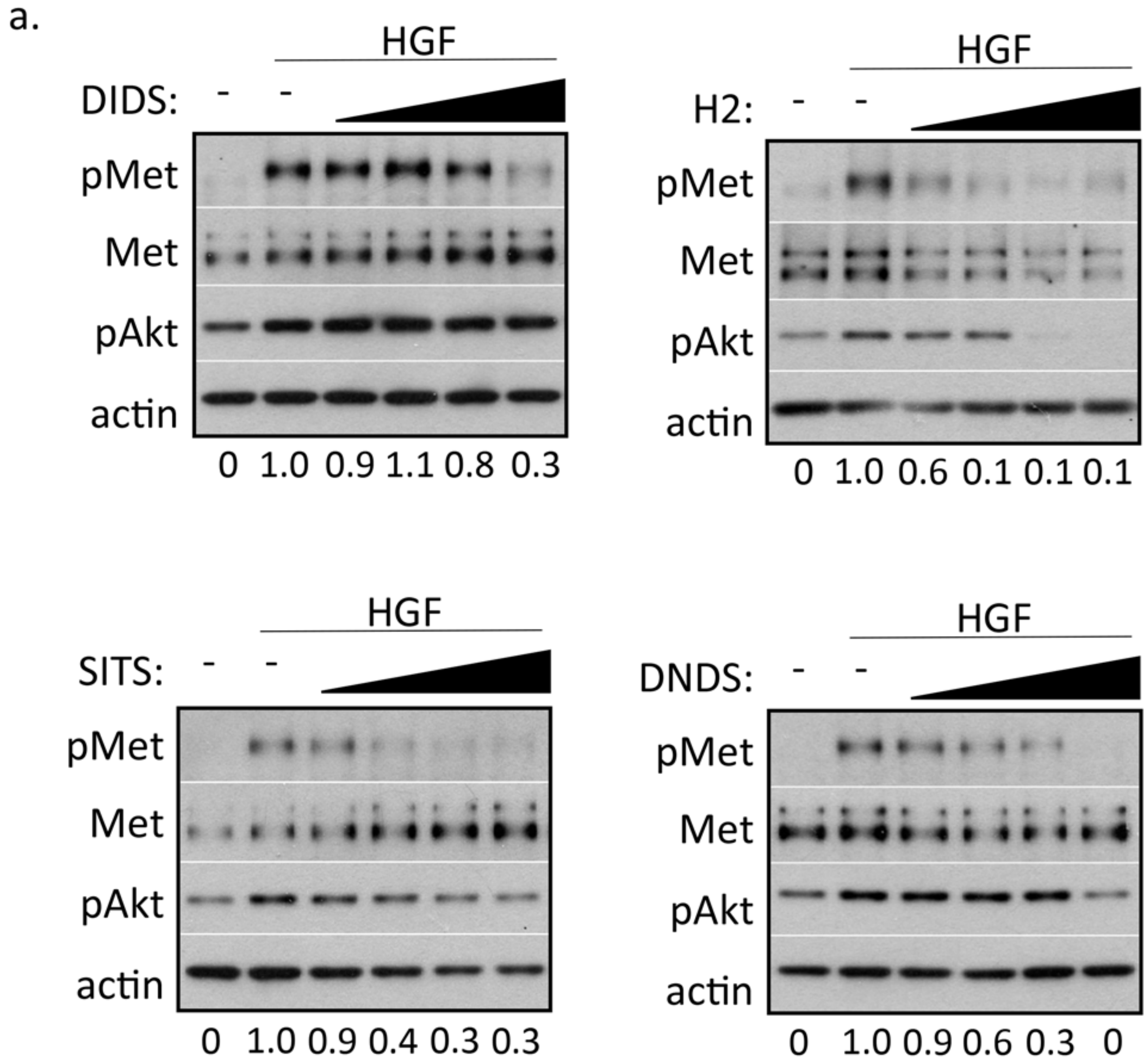

Supplementary Figure S1: Stilbene compounds reduce c-Met phosphorylation at similar concentrations in multiple cell lines. PC3 prostate cancer **a.** and MDA-MB-231 breast cancer

(*Continued*)

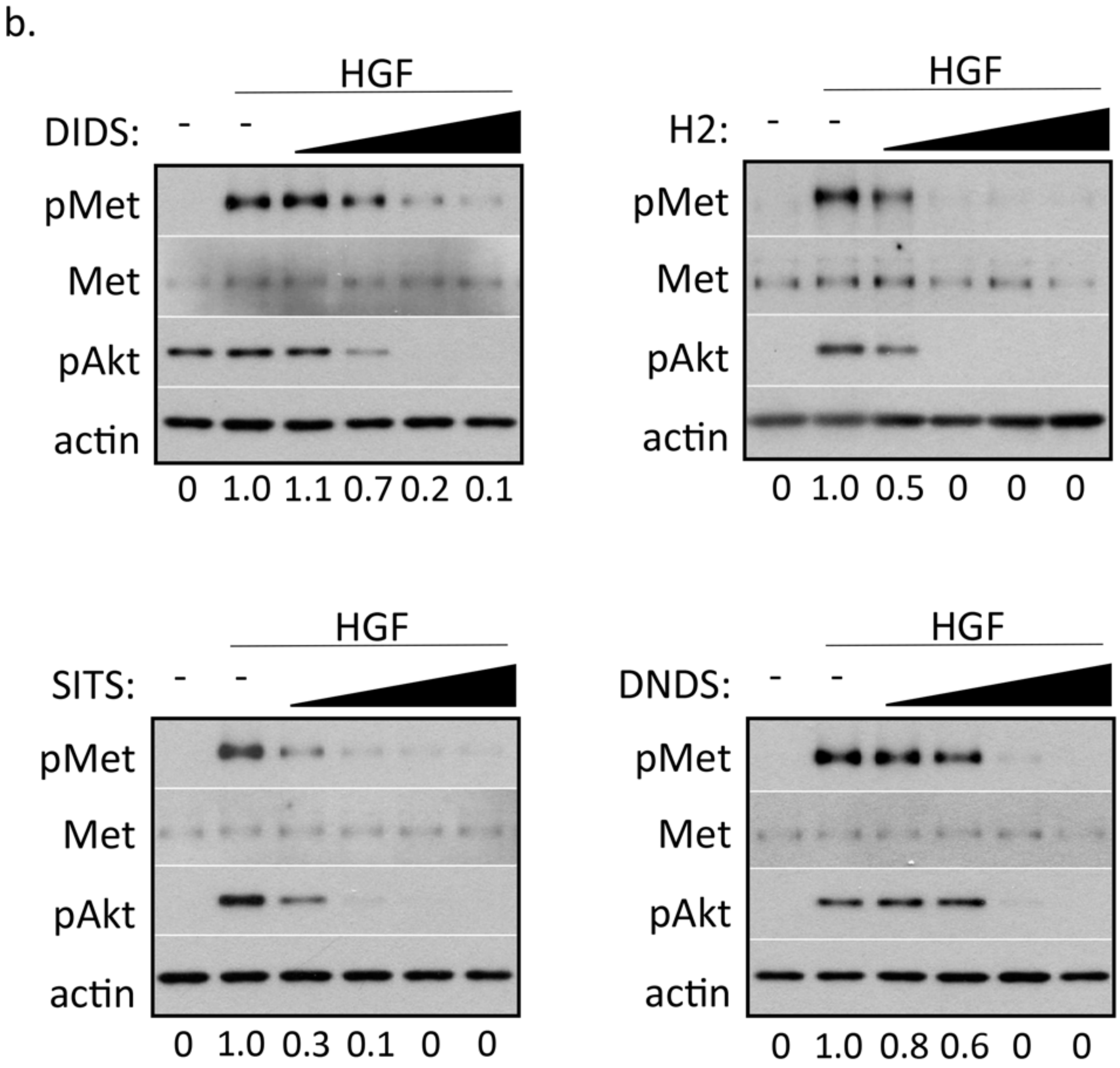

Supplementary Figure S1: (*Continued*) b. cells were treated with 33 ng/ml HGF for 20 minutes in serum-free media in the presence of 500 nM – 4 µM DIDS, 500 nM – 32 µM H2DIDS, 125 µM – 1 mM SITS, or 250 µM – 2 mM DNDS.

(*Continued*)

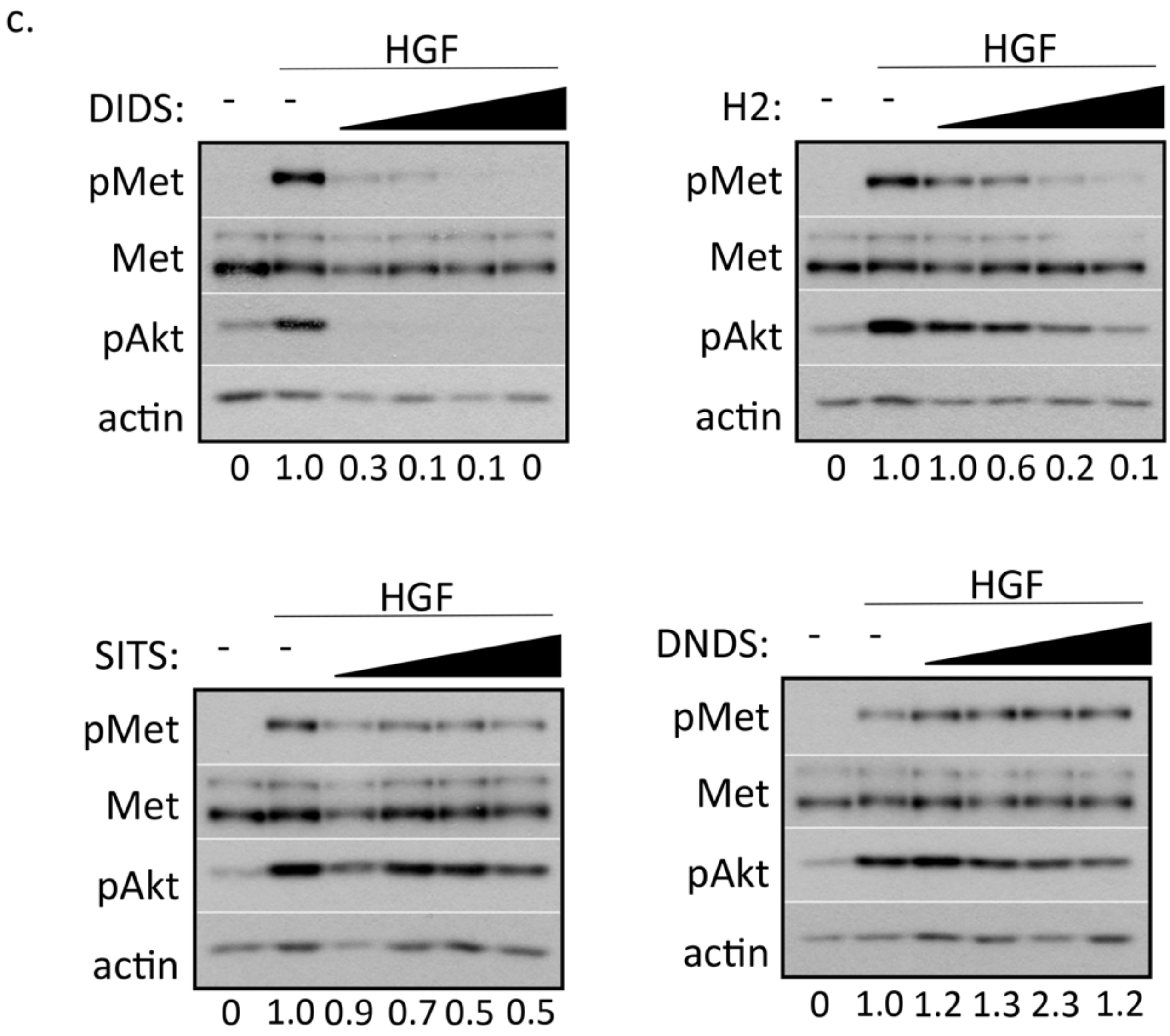

Supplementary Figure S1: (*Continued*) c. HCC1806 cells were treated similarly except 12.5 µM – 100 µM H2DIDS is shown. Western blot was used to analyze the indicated proteins. Densitometry shows changes in pMet compared to HGF control normalized to 1.

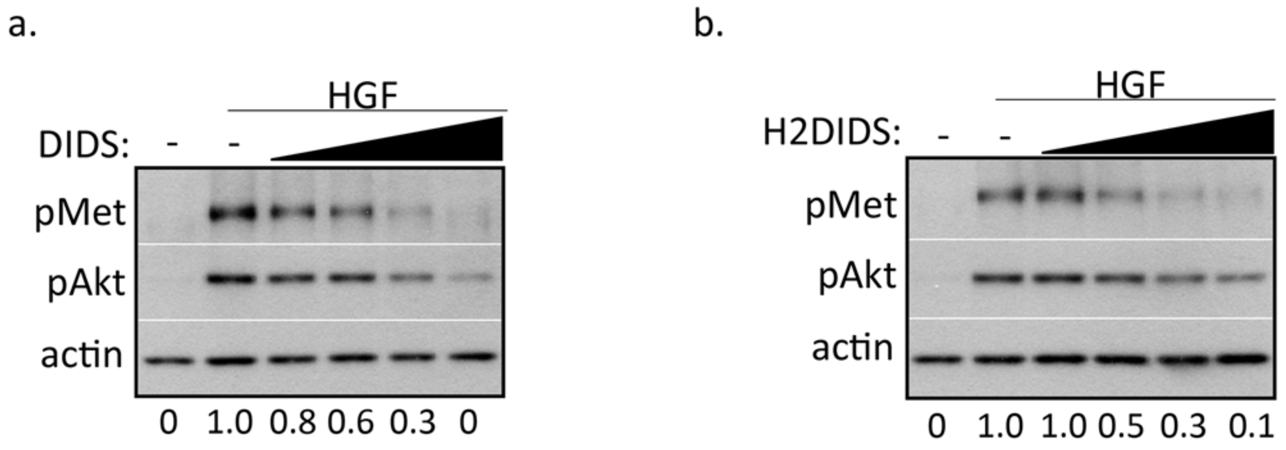

Supplementary Figure S2: Stilbene compounds reduce c-Met phosphorylation at similar concentrations in complete media. DU145 cells were treated with DIDS **a.** or H2DIDS **b.** at 1.3 µM, 3.8 µM, 11.3 µM, or 33.8 µM in the presence of 33 ng/ml HGF for 20 minutes in 10% FBS RPMI. Western blot was used to analyze the indicated proteins. Densitometry shows changes in pMet compared to HGF control normalized to 1.

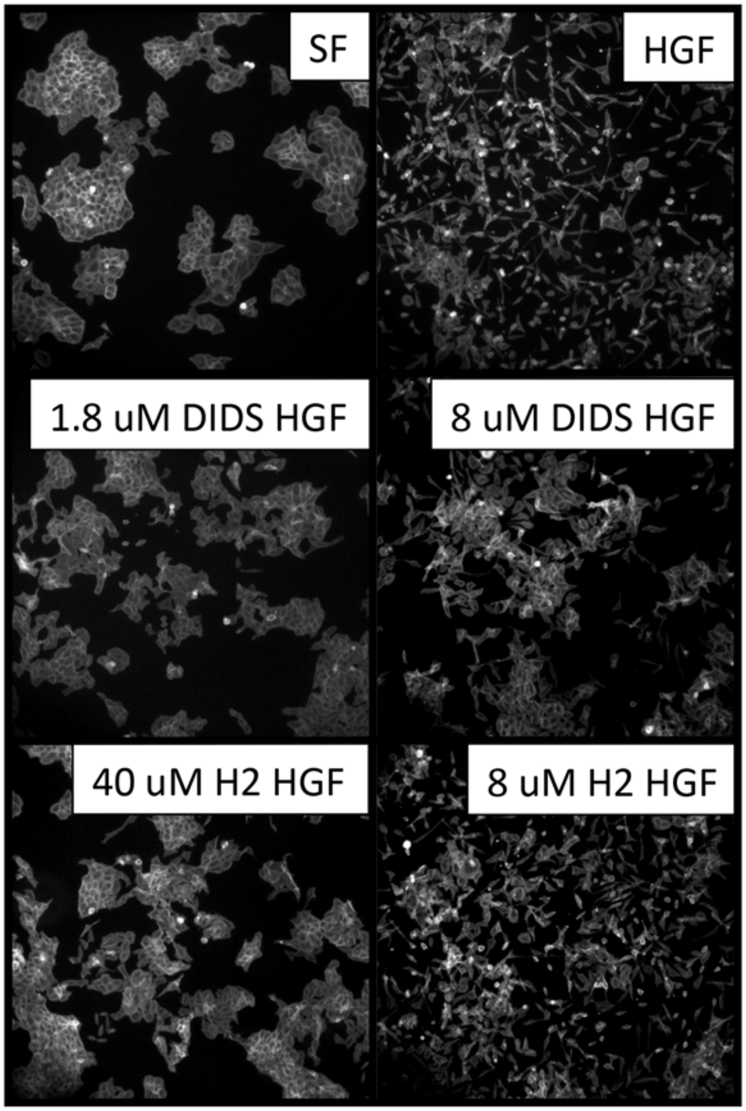

Supplementary Figure S3: DIDS and H2DIDS inhibit HGF-induced cell scattering in a dose-dependent manner. DU145 cells were treated with 33 ng/ml HGF overnight in the presence of 1.8 µM or 8 µM DIDS and 8 µM or 40 µM H2DIDS. Cells were fixed and stained for actin. Representative images are shown.

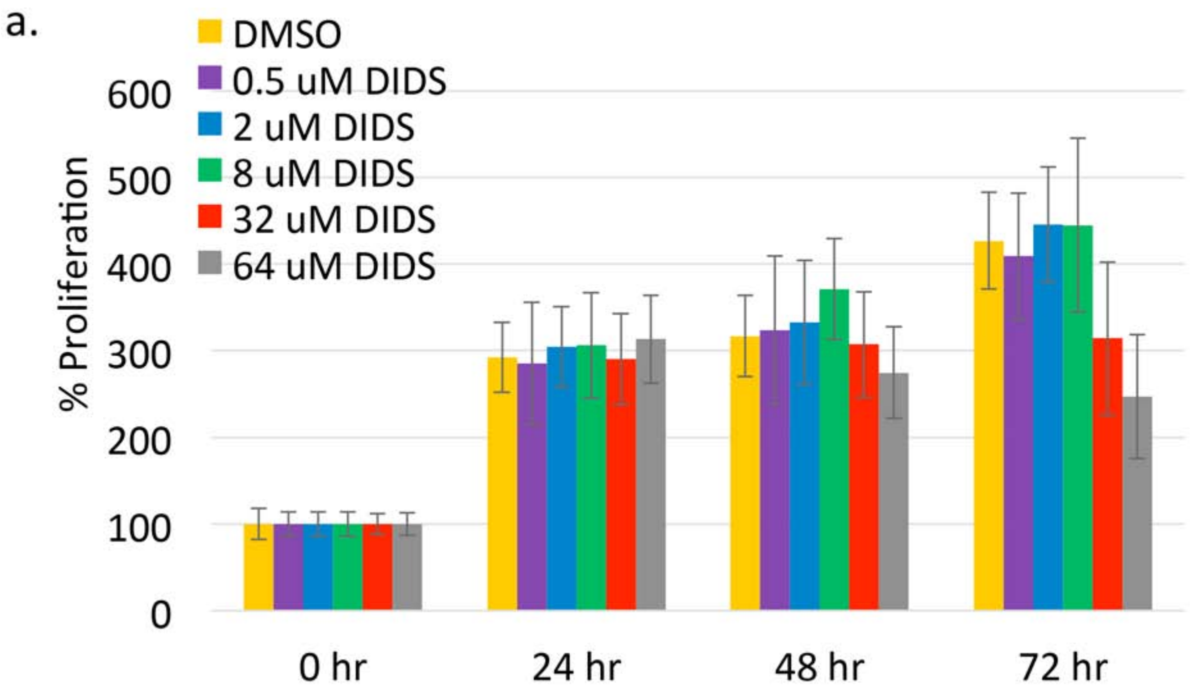

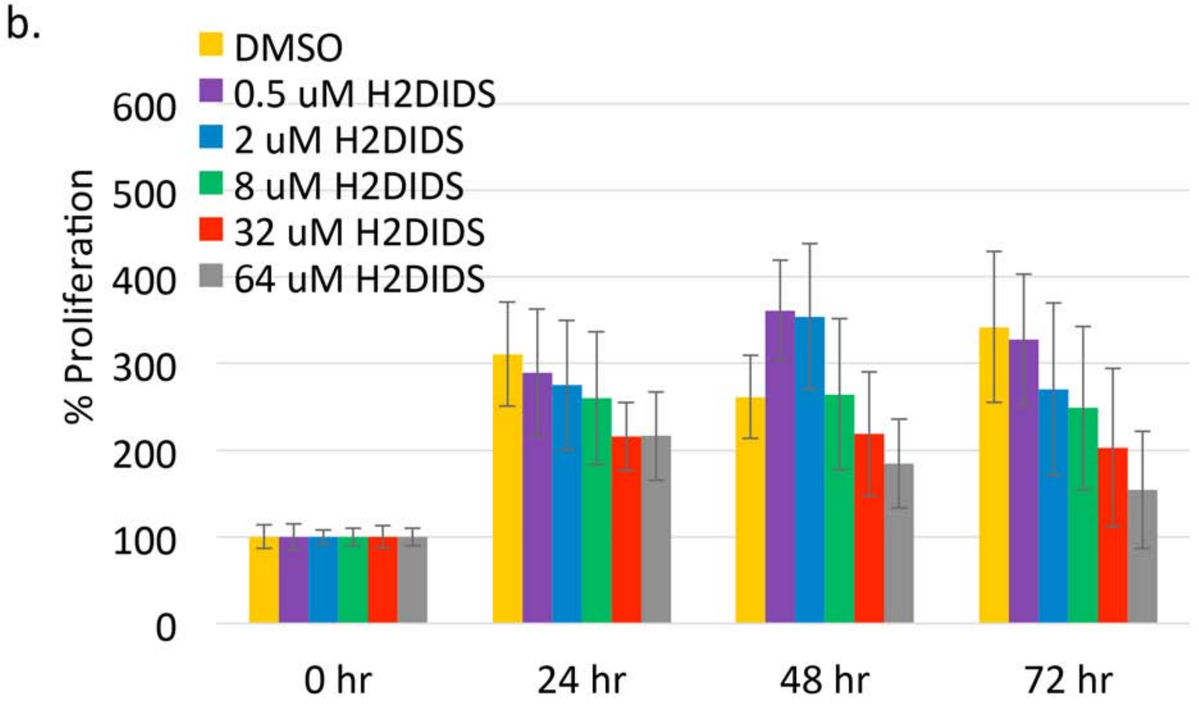

Supplementary Figure S4: DIDS and H2DIDS have minimal effects on 2D cell proliferation. Cells were treated with the indicated concentrations of DIDS **a, c.** or H2DIDS

(*Continued*)

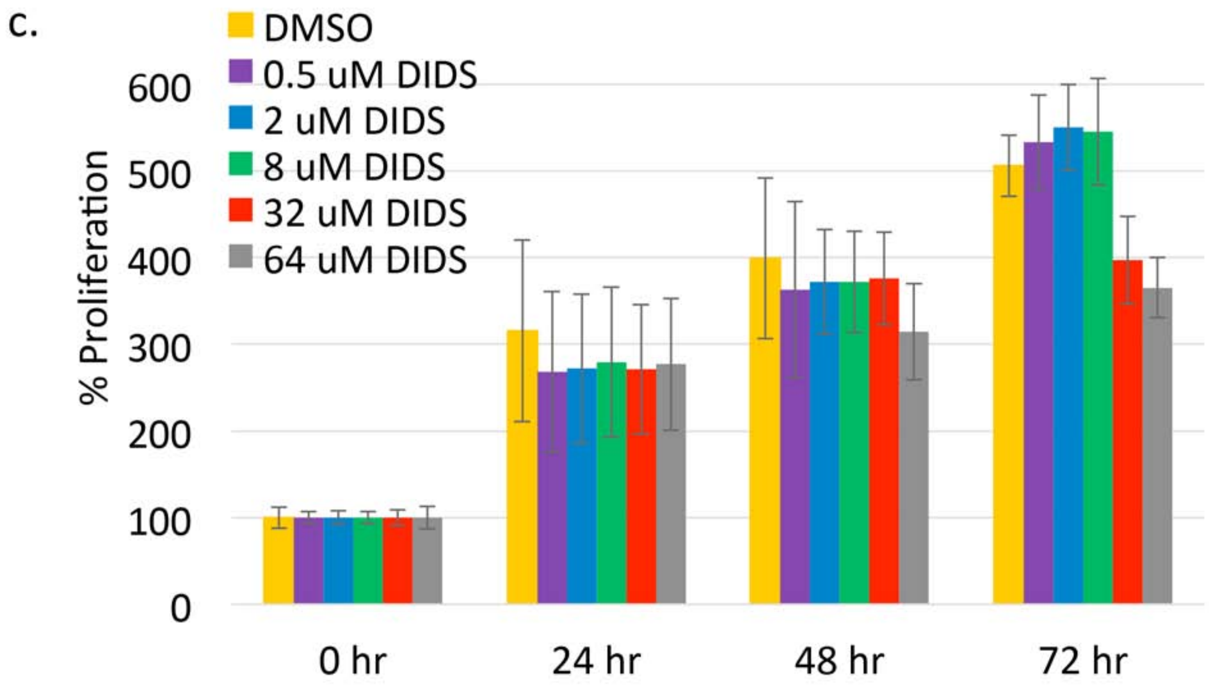

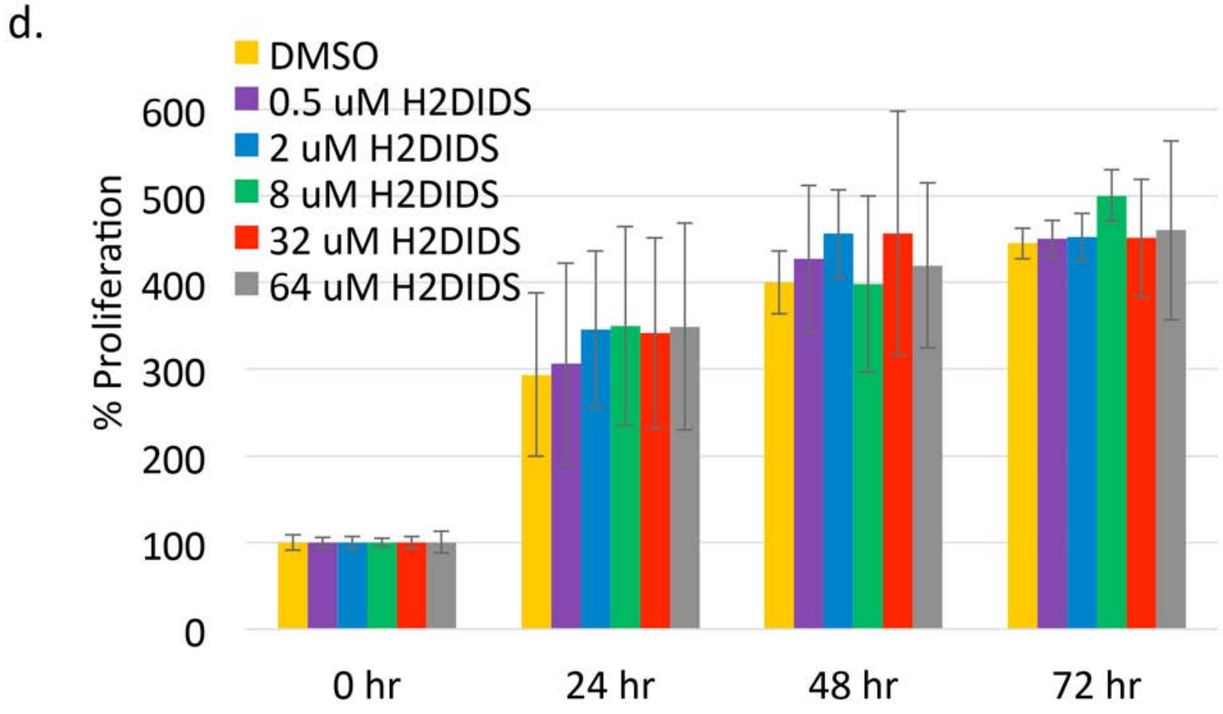

Supplementary Figure S4: (*Continued*) b, d. in serum-free media (a,b) or complete media (c,d) for the indicated times. Data are shown as mean ± S.E.M; n = 3.
